# The recurrent *TCF4* missense variant p.(Arg389Cys) causes a neurodevelopmental disorder overlapping with but not typical for Pitt‐Hopkins syndrome

**DOI:** 10.1111/cge.14206

**Published:** 2022-08-16

**Authors:** Bernt Popp, Thierry Bienvenu, Irina Giurgea, Julia Metreau, Cornelia Kraus, André Reis, Jan Fischer, María Palomares Bralo, Jair Tenorio‐Castaño, Pablo Lapunzina, Berta Almoguera, Fermina Lopez‐Grondona, Heinrich Sticht, Christiane Zweier

**Affiliations:** ^1^ Institute of Human Genetics University of Leipzig Medical Center Leipzig Germany; ^2^ Center of Functional Genomics Berlin Institute of Health at Charité ‐ Universitätsmedizin Berlin Berlin Germany; ^3^ INSERM U1266, Institut de Psychiatrie et de Neurosciences de Paris Université de Paris Paris France; ^4^ Département de Génétique Médicale INSERM Childhood Genetic Diseases, AP‐HP. Sorbonne Université, Hôpital Trousseau Paris France; ^5^ APHP, Service de Neurologie Pédiatrique Hôpital Universitaire Bicetre Le Kremlin‐Bicetre France; ^6^ Institute of Human Genetics Universitätsklinikum Erlangen, Friedrich‐Alexander‐Universität Erlangen‐Nürnberg Erlangen Germany; ^7^ Institute for Clinical Genetics University Hospital Carl Gustav Carus at the Technische Universität Dresden Dresden Germany; ^8^ INGEMM, Institute of Medical and Molecular Genetics Hospital Universitario La Paz, IDIPAZ Madrid Spain; ^9^ ITHACA European Reference Network Spain; ^10^ CIBERER, Centro de Investigación Biomédica en Red de Enfermedades Raras ISCIII Madrid Spain; ^11^ Department of Genetics and Genomics Fundación Jiménez Díaz University Hospital Madrid Spain; ^12^ Institute of Biochemistry Friedrich‐Alexander‐Universität Erlangen‐Nürnberg Erlangen Germany; ^13^ Department of Human Genetics Inselspital, Bern University Hospital, University of Bern Bern Switzerland

**Keywords:** intellectual disability, missense variant, neurodevelopmental disorder, Pitt‐Hopkins syndrome, PTHS, TCF4

## Abstract

*TCF4* haploinsufficiency by deletions, truncating variants or loss‐of‐function missense variants within the DNA‐binding and protein interacting bHLH domain causes Pitt‐Hopkins syndrome (PTHS). This neurodevelopmental disorder (NDD) is characterized by severe intellectual disability (ID), epilepsy, hyperbreathing and a typical facial gestalt. Only few aberrations of the N‐terminus of *TCF4* were associated with milder or atypical phenotypes. By personal communication and searching databases we assembled six cases with the novel, recurrent, *de novo* missense variant c.1165C > T, p.(Arg389Cys) in *TCF4*. This variant was identified by diagnostic exome or panel sequencing and is located upstream of the bHLH domain. All six individuals presented with moderate to severe ID with language impairment. Microcephaly occurred in two individuals, epilepsy only in one, and no breathing anomalies or myopia were reported. Facial gestalt showed some aspects of PTHS but was rather non‐specific in most individuals. Interestingly, the variant is located within the AD2 activation domain next to a highly conserved coactivator‐recruitment motif and might alter interaction with coactivator proteins independently from the bHLH domain. Our findings of a recurrent missense variant outside the bHLH domain in six individuals with an ID phenotype overlapping with but not typical for PTHS delineate a novel genotype–phenotype correlation for *TCF4*‐related NDDs.

## Graphical abstract

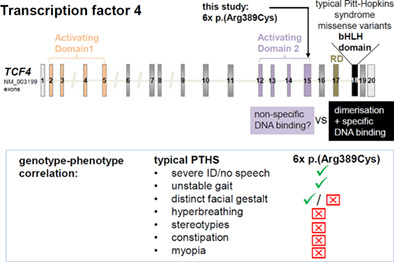

## 1 INTRODUCTION

Pitt‐Hopkins syndrome (PTHS, MIM #610954) was described in two unrelated individuals in 1978.^1^ In 2007, haploinsufficiency of *TCF4*, encoding a basic helix–loop–helix (bHLH) transcription factor binding to E‐box consensus motifs, was identified as the underlying molecular cause.^2^, ^3^

PTHS is characterized by severe intellectual disability (ID), lack of or significantly impaired speech development, behavioral issues, seizures, constipation, early‐onset myopia as well as a recognizable facial gestalt.^4^ The latter is characterized by a prominent nasal bridge, a beaked nose, prominence of the lower face and a wide mouth with a Cupid's bow shaped upper lip.^4^, ^5^ Additionally, a narrow forehead, thin lateral eyebrows, flared nasal alae, full cheeks and thickened/overfolded helices were delineated as distinctive.^6^

The majority of PTHS related aberrations affect exons 9–19 of *TCF4* (NM_003199.2; NM_001083962.2; 20 exons) and comprise large deletions, intragenic deletions, truncating and few elongating variants. About 20% of causative variants are missense variants located in the highly conserved bHLH domain, encoded by exon 18 (overview in Zollino et al.^6^).

Of note, several (likely) pathogenic *TCF4* variants have been associated with milder or non‐specific ID. While variants within the very N‐terminal exons 1–4 were found in individuals with mild ID,^7^ in some instances even inherited within a family,^8^, ^9^ loss‐of‐function variants in exons 7 and 8 seem to be associated with severe ID with or without typical features of PTHS.^7^, ^10^

We now report on six individuals harboring a recurrent missense variant (confirmed to be *de novo* in five individuals) upstream of the bHLH domain and presenting with rather non‐specific moderate to severe ID without or with only few facial aspects of PTHS.

## 2 METHODS AND RESULTS

By personal communication among colleagues, being contacted by one of the families, and by searching ClinVar,^11^ Varsome,^12^ and Decipher,^13^ we assembled six cases with the novel recurrent missense variant c.1165C > T, p.(Arg389Cys) in exon 15 (present in 46 isoforms in the human genome browser) of *TCF4* (NM_003199.3 or NM_001083962). The variant occurred *de novo* in five individuals. In one case, the father was not available. Consent to publish clinical and molecular data with or without clinical photographs was obtained from the parents or legal representatives.

Genetic analysis was performed by either diagnostic trio exome sequencing in three cases or sequencing clinical exome or panels of 56 or nine ID genes, respectively, in the others (Table 1). The p.(Arg389Cys) variant is not reported in gnomAD.^14^ It occurs three times in ClinVar^11^ with conflicting interpretation of pathogenicity (2× likely pathogenic, 1× with uncertain significance). One of the ClinVar cases is included in this study (SCV002011164.1). The variant affects a highly conserved amino acid and is predicted to be pathogenic by multiple *in silico* predictors (e.g., SIFT, Polyphen‐2, CADD).

**TABLE 1:** Clinical and molecular details of individuals with the *TCF4* variant c.1165C > T, p.(Arg389Cys) in exon 15 and comparison to typical Pitt‐Hopkins syndrome (PTHS)

	1	2	3	4	5	6	PTHS *n* = 150^6^
Test	Trio exome	56 genes ID panel	Sequencing of 9 ID genes	Trio exome	Trio exome	Clinical exome (4900 genes)	
Variant *de novo*	Yes	Yes	Yes	Yes	Yes	Unknown	
Gender	Female	Female	Male	Male	Female	Female	
Age last investigation	6 years 8 months	9 years 7 months	9 years 6 months	5 years	11 years 6 months	32 years 11 months	
Family history	Unremarkable	Parental consanguinity, otherwise unremarkable	Unremarkable	Short stature in the mother	Unremarkable	Unremarkable	
Normal birth measurements	Yes	Yes	Yes	Yes	NA	Yes	Usually normal
Growth/height	113 cm (P3‐P10)	Normal	150 cm (P90‐P97)	P2 at age 5.5 years	147.5 cm (P25‐P50)	156 cm (P3)	25% slow postnatal growth^4^
Weight	21.1 kg (P25‐P50)	Normal	33 kg (P50‐P75)	P15 at age 5.5 years	34.5 kg (P3‐P25)	45 kg	
OFC	52 cm (P50‐P75)	Normal	53 cm (P25‐P50)	P5 at age 5.5 years	52.5 cm (P25)	53 cm (P3)	19% microcephaly (up to 60%^4^)
Age of walking	31 months	26 months	20 months	24 months	20 months	26 months	95% gross motor delay
Age of first words	Not with 8 years	NA	5 years	Not with 5 years	3 years 6 months	Single word at 4 years	
Speech abilities	One single word, better perception	No sentences at 8 years, limited perception	Sentences, articulation problems, good perception	Undirected monosyllabic vocalization	Simple sentences, relatively good understanding	Absent speech	90% absent or very limited speech
ID	Severe	Moderate to severe (WPSI‐III: QIV, QIP, QIT 42–60)	Moderate	Severe	Moderate–severe (CI 46) at age 6 years	Severe	97% severe ID
Seizures	No	No	No	No	No	Yes, 12 years (carbamazepine)	37%
MRI	Normal	Normal	2× normal	Dysplastic corpus callosum	Normal	Normal	37.5% small corpus callosum, 29% wide ventricles
Muscular hypotonia	Yes	Yes, upper limbs and bucco‐facial with hypersalivation	No	No	Yes, first 3 years	Yes, 3–4 years	73% infantile axial hypotonia
Unstable gait	Yes	Yes	No	No	Until age 3 years	Yes	69% gait ataxia
Breathing anomalies	No	No	No	No	No	No	56%
Sleeping issues	No	No	No	No	No	No	
Behavioral issues	Little interaction, aggressive outbursts	Cheerful, attention deficit	No	Cheerful	No	No	60%
Stereotypic hand movements	No	No	No	No	No	No	59.5%
PTHS facial gestalt	No	No	No	No	No	partially	Yes
Narrow forehead	Mild	No	No	No	No	No	83%
Thin lateral eyebrows	No	No	No	No	No	No	76%
Wide nasal bridge/ridge/tip	No	No	Yes	Yes	Mild	Yes	91%
Flared nasal alae	No	No	No	No	No	Yes	79%
Full cheeks/prominent midface	No	No	No	No	No	No	85%
Wide mouth/full lips/cupid bow upper lip	No	No	Yes	No	No	Yes	95%
Thickened or overfolded helix	Yes	No	No	Yes	Simplified, ear pit	No	68%
Constipation	No	No	Mild	No	NR	No	81%
Myopia	No	NA	No, Mild hyperopia	No, hyperopia, strabism	No	No	54% myopia (early‐onset)
Hand anomalies (slender fingers and/or abnormal palmar creases)	Slender fingers	NA	No	No	No	Slender fingers	76%
Zollino et al. [1] PTHS score	4	3	1	2	2	8	>6 in all
Other anomalies	No	NR	No	No	Triangular face, diastemas of teeth, low‐set, simplified ears, gastroesophageal reflux	Ear lobe hypoplasia, multiple cavities, uterine myoma, early wrinkled skin	
Other exome results	No	No, only panel	No, only single genes	No	NR	No	
Other testing	Karyotyping, array and FraX normal	Karyotyping and FraX normal	Array normal, FraX normal, ALDH5A1, AHI1, TMEM67, CEP290, MED13, MED13L, NSUN2, TRAPPC9 sequencing normal, CFTR sequencing normal, metabolic screening normal	Karyotyping and array	karyotype, Fragile X, arrayCGH, metabolic work‐up all normal	Karyotype and metabolic work‐up, EEG, bone age. All normal	

*Notes*: *TCF4* (NM_003199.2); Shaded cells indicate aspects used for the clinical score as described by Zollino et al., (≥3/7 facial features: 4 points; severe ID with <5 words: 2 points; breathing anomalies: 2 points; myopia, constipation, hand anomalies, unstable gait: 1 point each; score ≥ 9 clinical confirmation of PTHS; score ≥ 6 PTHS possible; score < 6: insufficient evidence).

Abbreviations: NA, not available or not applicable; NR, not reported; P, percentile

**FIGURE 1:**
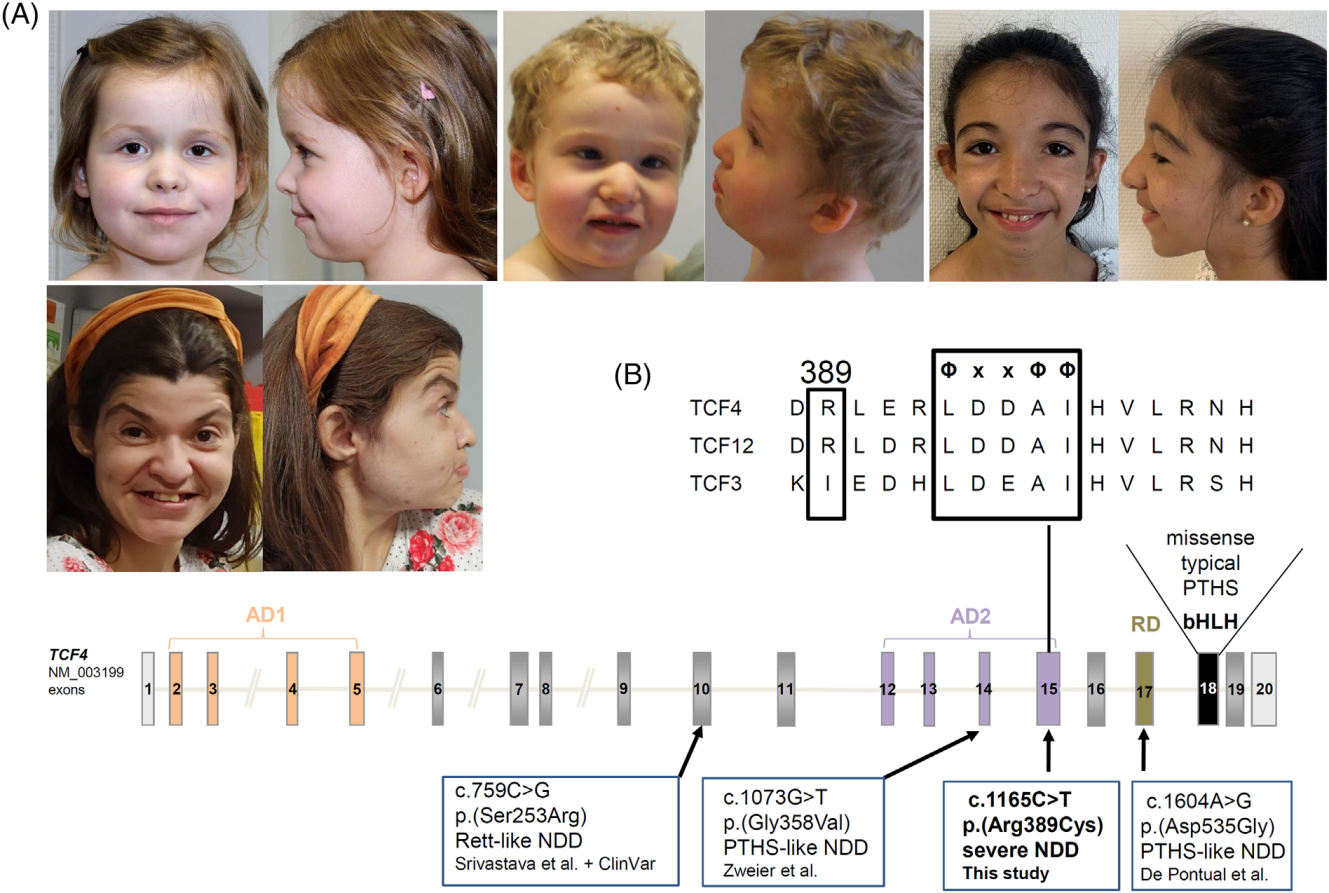
Missense variants in *TCF4*. (A) Facial gestalt of individuals 1, 4, 5, and 6, with I6 resembling Pitt‐Hopkins syndrome most. Subtle aspects such as overfolded helices, bitemporal narrowing in individual 1, a mildly beaked nose in individuals 4 and 6, enophthalmia in individuals 1, 4, and 6 and hypertelorism in individual 5. (B) Schematic drawing of *TCF4* with encoded domains and location of published and the novel missense variants and location and sequence of the conserved Φ‐x‐x‐Φ‐Φ activator‐recruitment motif within the AD2 domain. Non‐coding exons are depicted in light gray, coding exons in dark gray, domain encoding exons in color or in black. AD1 and AD2, activation domain 1 or 2; RD, repression domain; bHLH, basic helix–loop–helix domain [Colour figure can be viewed at wileyonlinelibrary.com]

Clinical details of the six affected individuals are summarized in Table 1. All had moderate to severe ID with lack of or severely impaired speech in four of them. Two individuals could speak in (simple) sentences and had good comprehension. Birth measurements and growth were unremarkable apart from microcephaly in two and mild growth delay in three individuals, of which one had a positive family history for short stature. Muscular hypotonia and/or an unstable gait were reported in four individuals, respectively, and behavioral issues in two individuals. Epilepsy occurred in one individual with an onset at age 12 years. The facial gestalt of I6 resembled PTHS most with deep‐set eyes, furrowing in the frontonasal ankle, a beaked nose with downturned nasal tip and a wide mouth. The other individuals showed single or few facial aspects of PTHS such as the beaked nose in I4 or overfolded helices in in several and overall a rather unspecific facial gestalt. None of the individuals has a protruding lower face or a Cupid bow's shaped upper lip, both typical for PTHS (Table 1, Figure 1).

## 3 DISCUSSION

The phenotype in the herewith reported six individuals with the recurrent c.1165C > T, p.(Arg389Cys) missense variant in *TCF4* includes moderate to severe ID, lack of or severely impaired speech, muscular hypotonia and minor facial dysmorphism. In none of these individuals, Pitt‐Hopkins syndrome was suspected before genetic testing. While severe ID with severely limited speech, muscular hypotonia and unstable gait overlap with PTHS, other typical and/or frequent aspects of PTHS such as breathing anomalies, constipation and myopia were not present. Severity of ID and speech impairment appeared more variable compared to PTHS as two of the individuals could speak in sentences.

As reflected by three clinical scoring systems, the most distinctive clinical aspect of PTHS is the recognizable facial gestalt.^6^, ^15^, ^16^ Whereas one of the individuals clearly resembled PTHS, this was less or not apparent in the other individuals. While single or few dysmorphism such as overfolded helices and/or a beaked nose were noted in most of them and do overlap with PTHS, the overall facial gestalt would, in our opinion, not allow a “facial diagnosis” of PTHS. Utilizing the most recently published clinical scoring system,^6^ none of the six individuals would reach a score allowing clinical diagnosis of PTHS and only one would reach a score indicating possible PTHS (Table 1). Interestingly, the oldest individual in this cohort at the age of 32 years showed the largest resemblance to PTHS. However, for speculating on the commonalities and differences between natural histories of typical PTHS and p.(Arg389Cys)‐associated NDD, a more prolonged observation and/or larger number of affected individuals would be required.

PTHS‐causing missense variants are usually located within the bHLH domain of *TCF4*, encoded by exon 18. This domain is crucial to form homodimers or heterodimers with other bHLH or HLH transcriptions factors and to bind DNA. It has been demonstrated that missense variants in the bHLH domain result in decreased transcriptional activation,^3^, ^17^, ^18^ which is in accordance with a loss‐of‐function and haploinsufficiency mechanism comparable to deletions or truncating variants.

Only few missense variants outside the bHLH domain of TCF4 have been reported. The *de novo* c.1073G > T, p.(Gly358Val) variant in exon 14 was identified in a 20‐year‐old individual within a PTHS cohort, presenting with severe ID, breathing anomalies, constipation, but without seizures and with a less typical facial gestalt.^5^ This variant did not result in significantly decreased activity in a transcriptional assay^5^ and also behaved similar to the wildtype in other assays.^18^ Another *de novo* missense variant, c.1604A > G, p.(Asp535Gly) in exon 17 was also identified within a PTHS cohort but clinical details were not provided.^17^ This variant was shown to have only minor functional effects *in vitro* but to change the ASCL1 and TCF4 heterodimer versus TCF4 homodimer binding preference.^18^ The variant c.759C > G, p.(Ser253Arg) in exon 10 was reported in a male individual with Rett‐like features but without clinical photographs.^19^ The same variant is contained three times in ClinVar as likely pathogenic and was shown to result in slightly reduced DNA binding and transactivation.^20^ In summary, so far reported missense variants outside the bHLH domain are associated with severe NDDs not completely typical for PTHS and behave more like the wildtype and different from PTHS‐related bHLH missense variants in *in vitro* assays.

Consequently, in at least three of the six individuals, the c.1165C > T, p.(Arg389Cys) variant was initially classified as of unknown significance due to its location outside the bHLH domain and to a rather unspecific neurodevelopmental phenotype not suggestive for PTHS. Only the recurrence of the identical variant in several individuals allowed its reclassification as pathogenic. This underlines the importance of careful interpretation and clinical follow‐up of exome sequencing results.

Interestingly, the c.1165C > T, p.(Arg389Cys) variant is located in the AD2 activation domain next to a highly conserved Φ‐x‐x‐Φ‐Φ motif (amino acid residues 393–397), which consists of three hydrophobic amino acids (Φ) that are spaced by two variable amino acids (x). This motif is conserved between TCF4 and TCF12 and mediates binding to the KIX domain of the transcriptional co‐activator CBP/p300.^21^ This variant might therefore impair interaction with coactivator proteins independently from the bHLH domain and in a less specific way as was demonstrated for the bHLH domain.^22^ Impaired heterodimer formation with HLH transcription factor ASCL1 and therefore impaired interaction with the ASCL1‐Phox‐Ret pathway was previously discussed to possibly underlie the autonomic dysregulation in PTHS in terms of breathing anomalies and constipation or Hirschsprung disease.^3^, ^17^ Indeed, only recently, loss of Phox2b‐expressing parafacial neurons have been reported in a *Tcf4* truncation (*Tcf4* ^tr/+^) mouse model which displayed hyperventilation and apnoea.^23^ Leaving the bHLH domain function and this specific interaction intact might explain the lack of breathing anomalies and constipation in the six individuals. Other clinical aspects that might be specifically related to the bHLH domain might include craniofacial abnormalities, seizures, and myopia.

To conclude, our findings of a recurrent missense variant upstream of the bHLH domain in six individuals with rather non‐specific, severe ID overlapping with but not typical for PTHS delineates a novel genotype–phenotype correlation for *TCF4*‐related NDDs.

## AUTHOR CONTRIBUTIONS

Bernt Popp, Thierry Bienvenu, Irina Giurgea, Julia Metreau, Cornelia Kraus, André Reis, Jan Fischer, María Palomares Bralo, Jair Tenorio Castano, Pablo Lapunzina, Berta Almoguera, Fermina Lopez‐Grondona, and Christiane Zweier collected mutational and clinical data. Heinrich Sticht performed structural modeling. Bernt Popp and Christiane Zweier wrote the manuscript, which was read and revised by all co‐authors.

## CONFLICT OF INTEREST

The authors declare no conflict of interest.
